# Detrusor sphincter dyssynergia: can a more specific definition distinguish between patients with and without an underlying neurological disorder?

**DOI:** 10.1038/s41393-021-00635-3

**Published:** 2021-05-07

**Authors:** Oliver Gross, Lorenz Leitner, Maria Rasenack, Martin Schubert, Thomas M. Kessler

**Affiliations:** 1 Department of Neuro-Urology, Balgrist University Hospital, University of Zürich, Zürich, Switzerland; 2 Spinal Cord Injury Center, Balgrist University Hospital, University of Zürich, Zürich, Switzerland

**Keywords:** Spinal cord diseases, Neurogenic bladder

## Abstract

**Study design:**

Cross-sectional study.

**Objectives:**

To evaluate if specific definitions of detrusor sphincter dyssynergia (DSD) might distinguish between individuals with spinal cord injury (SCI) and those with no underlying neurological disorder (NO ND).

**Setting:**

Single tertiary university SCI center.

**Methods:**

A series of 153 individuals, 81 with traumatic SCI and 72 with NO ND, were prospectively evaluated and included in this study. All individuals underwent a clinical neuro-urological examination, a neurophysiological work-up and a video-urodynamic investigation and were diagnosed with DSD as defined by the International Continence Society (ICS). We determined the DSD grades/types according to the classifications by Yalla (grade 1–3), Blaivas (type 1–3) and Weld (type 1–2). Distribution of the DSD grades/types were compared between SCI and NO ND individuals. Associations between the various DSD grades/types and clinical parameters, such as risk factors for upper urinary tract damage (all individuals) or lower extremity motor scores, SCI injury levels and severity scores (only SCI group), were assessed.

**Results:**

The distribution of all DSD types were similar between groups (*p* > 0.05). None of the DSD classifications allowed risk assessment for upper urinary tract damage. A significant association between DSD type and other clinical parameters could not be found (*p* > 0.05).

**Conclusions:**

None of the investigated DSD definitions can distinguish between patients with SCI and with NO ND. The more complex DSD classifications by Yalla, Blaivas or Weld cannot compete with the ICS binary yes-no definition which is pragmatic and straightforward for managing patients in daily clinical practice.

**Sponsorship:**

None.

## Introduction

Detrusor sphincter dyssynergia (DSD), also known as detrusor striated-sphincter dyssynergia or detrusor external-sphincter dyssynergia, is a urodynamic observation defined by the International Continence Society (ICS) as a detrusor contraction concurrent with an involuntary contraction of the urethral and/or periurethral striated muscle [1, 2]. DSD is pathophysiologically considered to be a neurological problem impairing the ability of the pontine micturition center or its pathways to co-ordinate the function of the sacral lower urinary tract (LUT) spinal centers [3, 4] and it typically occurs in individuals with suprasacral spinal lesion due to spinal cord injury (SCI), spina bifida or multiple sclerosis. Nevertheless, DSD can also be observed in patients without spinal lesions and even healthy volunteers [5].

DSD can result in voiding difficulties and incomplete bladder emptying and in combination with detrusor overactivity in dangerously high pressures and morphological changes of the lower and upper urinary tract eventually leading to end-stage renal disease [6]. In 1977, Yalla et al. [7] suggested a grading to determine the degree of DSD and its influence on voiding function. An adapted classification, focusing on the electromyographic (EMG) profile, was published by Blaivas et al. [8] in 1981. Weld et al. [9] proposed in 2000 a categorization between an intermittent or continuous sphincter contraction. However, none of the classifications allowed a risk assessment for individuals with SCI. Hence, the current definition of DSD recommended by the ICS is a binary yes-no variable [1].

The primary aim of this study was to evaluate if a more specific DSD definition might distinguish between individuals with SCI and those with no underlying neurological disorder (NO ND) diagnosed with a DSD and thereby influence the management in daily clinical practice. Secondary outcomes included the association of different DSD definitions and clinical parameters such as risk factors for upper urinary tract damage or SCI severity.

## Methods

### Participants

From September 2006 to February 2020, a series of 153 individuals with traumatic SCI leading to tetraplegia or paraplegia or individuals with NO ND were prospectively evaluated at the Department of Neuro-Urology, Balgrist University Hospital, Zürich, Switzerland, a tertiary referral center for patients with any kind of lower urinary tract dysfunction (LUTD) and the SCI Center, Balgrist University Hospital, Zürich, Switzerland. All individuals were urologically treatment naïve, had a complete neuro-urological, neurological, and neurophysiological examination. Only individuals with a DSD diagnosed by video-urodynamic investigation (VUDI) with synchronous EMG and fluoroscopy were included. VUDI had to be performed not earlier than 3 months after SCI in a sitting position considering the spinal shock phase [6, 10] and allowing a physiological voiding position. In individuals without signs for an underlying neurological disorder in the neurological and neurophysiological work-up, the time of the examination corresponded to the time of presentation in our tertiary referral center. The study including all experimental protocols were approved by the local ethics committee (Kantonale Ethikkommission Zürich). Informed consent was obtained from all participants. Methods were carried out in accordance with the relevant clinical guidelines provided by the American Spinal Injury Association (ASIA), European Associations of Urology and ICS. All definitions and units are according to the standards recommended by the ICS [1].

### Investigations

All participants underwent neuro-urological assessment [6] consisting of medical history, examination of urogenital sensation, bulbocavernosus reflex (performed by squeezing the clitoris or glans during digito-rectal examination) and pelvic floor EMG, anal reflex, anal sphincter tone and anal squeeze response.

The neurological examination was performed according to the International Standards for Neurological Classification of Spinal Cord Injury (ISNCSCI) [11–13], an established neurological assessment developed and published by ASIA, by trained physicians with certified experience in SCI examination and classification, after a specific centralized training program [14]. ISNCSCI lower extremity motor score (LEMS) and light-touch and pinprick scores, allowing the definition of neurological level, motor level and sensory level and classification with the ASIA Impairment Scale (AIS) [15] in five different grades of severity (from A = complete lesion to E = normal sensation and motor function in all segments), were taken into consideration for further evaluation.

Neurophysiological examination consisted of somatosensory evoked potentials (SEPs) and motor evoked potentials (MEPs). Technical details of neurophysiological examinations have been published previously [16, 17]. SEP recordings were obtained bilaterally following stimulation of the tibial nerves. MEPs were obtained bilaterally following transcranial magnetic stimulation of the corresponding cortical motor area from anterior tibial muscles. The neurophysiological examination confirmed the lesion of spinal tracts in individuals with SCI. All other individuals were judged as having NO ND as they presented no motor or sensory deficits in the neurological exam and showed normal evoked potential studies.

VUDI was performed according to “Good Urodynamic Practice” recommended by the ICS [18, 19], in a sitting position, using a multichannel urodynamic system and comprised same session repeat filling cystometry and pressure-flow study. For intravesical and rectal pressure recordings, a water-perfused 6-Fr double lumen transurethral catheters and common rectal balloon catheter were used, respectively. The infusion rate during the filling cystometry was between 20 and 30 mL/min. Pelvic floor EMG data was recorded using two surface electrodes placed bilaterally on the perineum close to the external anal sphincter. Involuntary contraction of the urethral and/or periurethral striated muscle, i.e., elevated EMG signal, during detrusor contraction, was defined as DSD, in line with the ICS definition [1] and diagnosed in all included individuals. All VUDI were re-assessed independently by two experienced consultants in neuro-urology according to different DSD classifications:

Yalla et al. (1977) [7]:

- Grade 1: high intravesical voiding pressures resulting from the resistance offered by the semi-compliant striated sphincter
- Grade 2: either inappropriate or clonic striated sphincter contractions resulting in interrupted voiding
- Grade 3: non-voiding secondary to sustained spasticity of the external sphincter with complete closure of the outlet

Blaivas et al. (1981) [8]:

- Type 1: crescendo increase in EMG activity that suddenly relaxes at peak of detrusor contraction
- Type 2: clonic sphincter contractions interspersed throughout detrusor contraction
- Type 3: sustained sphincter contraction that persists throughout bladder contraction

Weld et al. (2000) [9]:

- Type 1: intermittent sphincter contraction interspersed throughout detrusor contraction
- Type 2: continuous sphincter contraction that persists throughout bladder contraction

ICS definition (2002) [1]: Involuntary contraction of the urethral and/or periurethral striated muscle, i.e., elevated EMG signal, during detrusor contraction (yes/no).

To adhere to original publications, we use the terms “grade” for the classification by Yalla [7] and “type” for the Blaivas [8] and Weld [9] classification system. VUDI findings were categorized regarding occurrence of maximum storage detrusor pressure at urethral leakage ≤40cmH2O or >40cmH2O (or only maximum storage detrusor pressure in case of no urethral leakage), a value expected as indicator for increased risk for upper urinary tract deterioration [6, 20, 21].

### Statistical analyses

Data distribution was tested by Q-Q plots. Normally distributed data are presented as mean ± standard deviation (SD), skewed data as median (25% and 75% percentiles). Comparing unrelated samples, the unpaired *t*-test was used for approximately normally distributed data and the Mann–Whitney U test for skewed data.

For comparisons of individuals with SCI and NO ND and the distribution of DSD according to the different classifications an omnibus test for goodness-of-fit model by the Freeman–Halton extension of the Fisher’s exact probability test (for a two-rows by three-columns contingency table (Yalla grade 1–3, Blaivas type 1–3)) and the Fisher’s exact test (Weld type 1–2) were used.

To evaluate the association between the DSD grade/type for the different classifications and detrusor pressure ≤40cmH2O versus >40cmH2O (i.e., risk for upper urinary tract damage) a logistic regression was performed using Yalla grade 1; Blaivas type 1 and Weld type 1 as reference parameters. Pearson chi-square test was applied to evaluate the relationship between DSD classification and AIS grades of severity (i.e., completeness) of the injury, DSD classification and LEMS were assessed by Spearman correlation.

Statistical analyses were applied using IBM’s Statistical Package for the Social Sciences (SPSS) Version 24.0 (IBM SPSS Statistics for Windows, V24.0, Armonk, NY, USA) with *p* < 0.05 considered statistically significant.

## Results

Characteristics of all 153 individuals included in the study are shown in Table 1. There was no statistically significant difference in terms of age of the participants (*p* = 0.155), but the proportion of men was greater in the SCI compared to the NO ND group (80% versus 60%). The median duration between SCI and VUDI was 3 (3–3) months.

**Table 1:** Patients’ characteristics.

	SCI (*n* = 81)	NO ND (*n* = 72)	*p* value
Age [year]	50 ±20	44 ±14	0.155
Sex			
Male [n] (%)	65 (80%)	43 (60%)	0.005
Female [n] (%)	16 (19%)	29 (40%)	
Type of bladder emptying			
Spontaneously [n] (%)	21 (26%)	61 (85%)	<0.001
Intermittent catheterization [n] (%)	11 (14%)	10 (14%)	0.64
Indwelling catheter [n] (%)	49 (60%)	1 (1%)	<0.001
Characteristics for individuals with SCI			
Tetraplegic [n] (%)	49 (61%)	N/A	N/A
Paraplegic [n] (%)	32 (39%)	N/A	N/A
AIS grades of severity			
Grade A [n] (%)	29 (36%)	N/A	N/A
Grade B [n] (%)	6 (7%)	N/A	N/A
Grade C [n] (%)	18 (22%)	N/A	N/A
Grade D [n] (%)	28 (35%)	N/A	N/A
Grade E [n] (%)	0 (0%)	N/A	N/A
Months between SCI and VUDI	3 (3/3)	N/A	N/A
LEMS [score value]	17 (16/49)	50 (50/50)	N/A

Data are presented as mean and standard deviation or median (25% percentiles/75% percentiles) as appropriate. Percentages may not total 100 due to rounding.

*SCI* spinal cord injury, *NO ND* individuals without signs for underlying neurological disorder, *LEMS* lower extremity motor score, *N/A* not applicable, *AIS* American Spinal Cord Injury Association impairment scale, *VUDI* video-urodynamic investigation.

Omnibus test of goodness-of-fit did not reveal a general significance in the overall hypothesis regarding distribution of Yalla grade 1–3 (*p* = 0.059) and Blaivas type 1–3 (*p* = 0.478) between the SCI and NO ND group, hence, no post-hoc Fisher’s exact text for the different subcategories were performed. There was no statistically significant difference between the two groups for distribution of Weld type 1 and 2 (*p* = 0.195) (Table 2).

**Table 2:** Distribution of DSD according the different classifications.

	SCI	NO ND	*p* value
Yalla grade 1 [n] (%)	1 (1%)	6 (8%)	0.059
Yalla grade 2 [n] (%)	51 (63%)	36 (51%)	
Yalla grade 3 [n] (%)	29 (36%)	30 (41%)	
Blaivas type 1 [n] (%)	18 (22%)	13 (18%)	0.478
Blaivas type 2 [n] (%)	36 (44%)	28 (39%)	
Blaivas type 3 [n] (%)	27 (34%)	31 (43%)	
Weld type 1 [n] (%)	39 (48%)	27 (38%)	0.195
Weld type 2 [n] (%)	42 (52%)	45 (62%)	

*DSD* detrusor sphincter dyssynergia, *SCI* spinal cord injury, *NO ND* individuals without signs for underlying neurological disorder.

None of the DSD classifications allowed risk assessment comparing individuals with a maximum storage detrusor pressure ≤40cmH2O versus >40cmH2O (i.e., risk for upper urinary tract damage) (Table 3).

**Table 3:** Relationship of DSD classification and maximum storage detrusor pressure >40cmH2O (i.e., risk factor for upper urinary tract damage).

DSD classification	SCI (*n* = 81)			NO ND (*n* = 72)			All individuals (*n* = 153)		
	OR	95% CI	*p* value	OR	95% CI	*p* value	OR	95% CI	*p* value
Yalla grade 1	Ref. ^a^	–	–	Ref. ^a^	–	–	Ref. ^a^	–	–
Yalla grade 2	0.00	0.00	1	0.00	0.00	1	0.00	0.00	1
Yalla grade 3	1.34	0.53–3.41	0.53	1.53	0.49–4.88	0.46	1.51	0.74–3.07	0.26
Blaivas type 1	Ref. ^a^	–	–	Ref. ^a^	–	–	Ref. ^a^	–	–
Blaivas type 2	1.46	0.44–4.84	0.54	1.56	0.31–7.78	0.59	1.66	0.66–4.18	0.28
Blaivas type 3	0.93	0.33–2.56	0.88	2.08	0.59–7.34	0.26	1.38	0.64–2.98	0.42
Weld type 1	Ref. ^a^	–	–	Ref	–	–	Ref. ^a^	–	–
Weld type 2	0.93	0.38–2.24	0.87	1.95	0.63–6.00	0.25	1.34	0.68–2.65	0.4

Ref. ^a^ Yalla grade 1; Blaivas type 1 and Weld type 1 were used as reference parameter for the logistic regressions for each of the DSD classifications. Patients with vesico-uretero-renal reflux (*n* = 2) (e.g., Fig. 2a) were classified according the measured maximum despite a potential underestimation of the real maximum detrusor storage pressure.

*DSD* detrusor sphincter dyssynergia, *OR* odds ratio, *CI* confidence intervals, *SCI* spinal cord injury, *NO ND* individuals without signs for underlying neurological disorder.

**Fig. 1:**
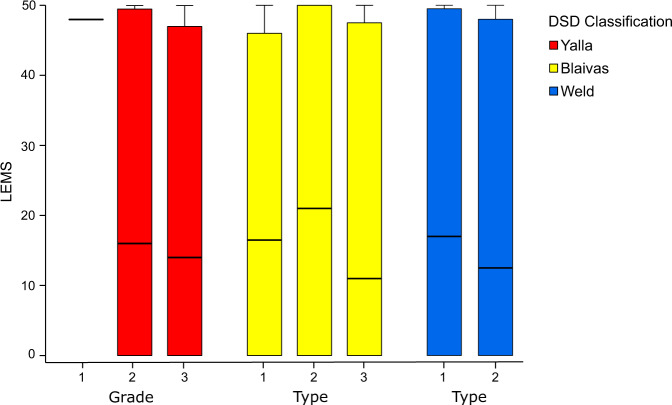
Boxplots for distribution of LEMS and DSD classifications provided by Yalla, Blaivas and Weld compared with LEMS. Individuals without signs for underlying neurological disorder (NO ND) were excluded from this analysis as they had by definition a LEMS of 50, no significant correlation between LEMS and DSD classification was found (*p* > 0.05). DSD = detrusor sphincter dyssynergia, SCI = spinal cord injury, LEMS = lower extremity motor score.

SCI within-group analyses did not reveal a significant association (*p* > 0.05) between DSD classification and LEMS (Fig. 1) or AIS grades of severity (i.e., completeness) of the injury.

**Fig. 2:**
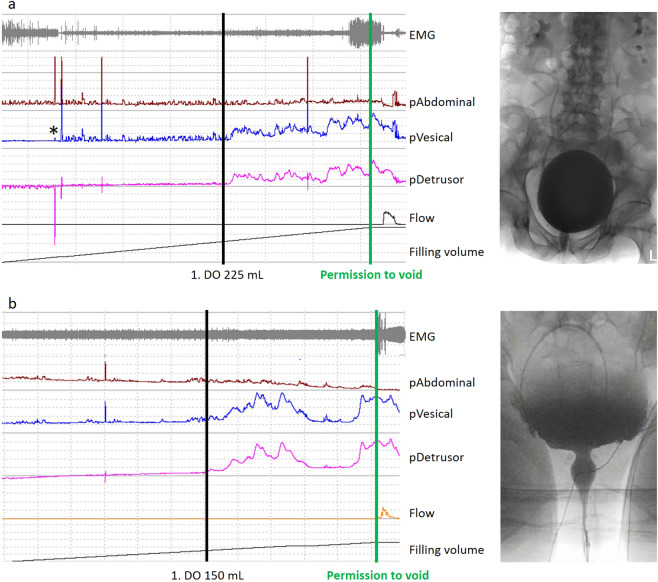
Typical video-urodynamic findings in an individual with SCI and NO ND included in the study. **a** With SCI: VUDI findings (3 months after injury) of a 75-year-old man with incomplete traumatic tetraplegia (AIS D, lesion level C3) and a LEMS of 50, voiding spontaneously. Neuro-urological and neurophysiological examinations support the diagnosis. First detrusor overactivity at 225 mL with a maximum detrusor pressuring during filling cystometry of 62 cmH2O, the maximum bladder capacity is 530 mL, no post-void residual. DSD is diagnosed by EMG as well as by fluoroscopy. DSD is classified as grade 2, type 1 and type 2 according to the classifications by Yalla, Blaivas and Weld, respectively. Fluoroscopy shows a bilateral vesico-uretero-renal reflux, a finding that could lead an underestimation of the real maximum detrusor storage pressure. **b** With NO ND: VUDI findings of a 38-year-old women with LUTS (urgency, frequency and urgency incontinence) of unknown origin, voiding spontaneously. Neuro-urological and neurophysiological examinations are inconspicuous. First detrusor overactivity at 150 mL with a maximum detrusor pressuring during filling cystometry of 78 cmH2O, the maximum bladder capacity is 220 mL, post-void residual 20 mL. DSD is detected in the EMG, in the fluoroscopy and in the uroflowmetry. DSD is classified as grade 2, type 2 and type 1 according to the classifications by Yalla, Blaivas and Weld, respectively. VUDI = video-urodynamic investigation, LUTS = lower urinary tract symptoms, DO = detrusor overactivity, DSD = detrusor sphincter dyssynergy, SCI = spinal cord injury, NO ND = individual without signs for underlying neurological disorder, EMG = electromyography, LEMS = lower extremity motor score, AIS = American Spinal Cord Injury Association impairment scale. *Catheter adjustment after poor transmission of the vesical pressure.

Typical VUDI findings of an individual with SCI and with NO ND are provided in Fig. 2.

## Discussion

### Main findings

The currently most widely used DSD definition by the ICS is simple, that means DSD is characterized as involuntary contraction of the urethral and/or periurethral striated muscle, i.e., elevated EMG signal, during detrusor contraction [1]. This definition, introduced already in 2002 [1], has stood the test of time and our findings indicate that more sophisticated classifications by Yalla, Blaivas or Weld cannot seriously compete with the ICS binary yes-no definition which is pragmatic and straightforward, even in the individualized daily management of challenging neurogenic LUTD in complex neuro-urological patients. However, none of the DSD classifications can distinguish between patients with SCI and without an underlying neurological disorder and can also not detect patients with a maximum storage detrusor pressure ≤40cmH2O versus >40cmH2O, a risk factor for upper urinary tract damage or for a more severe neuro-urological course. Thus, urodynamic findings need to be considered in the context of LUT symptoms and neuro-urological signs to allow an appropriate patient management in daily clinical practice.

### Findings in the context of existing evidence

DSD is typically found in neurological patients with a suprasacral spinal cord lesion, mostly due to SCI, spina bifida or multiple sclerosis. In neurologically normal individuals, the terms detrusor sphincter dyscoordination, dysfunctional voiding or non-relaxing urethral sphincter obstruction instead of DSD are commonly used, especially in German speaking countries. However, it is of questionable value from a clinical perspective to apply different terms for the same urodynamic observation [5] taking into consideration, that (I) patients will not be treated according to a definition but rather based on their LUT symptoms, (II) that the urodynamic phenomenon of a detrusor contraction is only considered by the DSD definition but not by the other terms and (III) that an inappropriate terminology could downplay a serious condition and delay adequate treatment, especially as an underlying neurological disorder cannot be ruled out by a urodynamic examination.

In line with Weld et al. [9], we found no significant relationship of the type of DSD with symptom severity. Voiders and individuals relying on catheterization as well as individuals with and without potential risk for upper urinary tract damage suffered from both type 1 and type 2 DSD. Schurch et al. suggested a correlation between AIS grades of severity and the Blaivas DSD classification [22]. This is in contrast to our findings and probably due to relevant differences in the patient cohorts, for instance the duration between SCI and VUDI. In the present study, we could not detect a significant relationship between DSD classifications and LEMS what is supported by Bellucci et al. reporting a similar occurrence of DSD in ambulatory and non-ambulatory individuals with SCI [23].

Kirby et al. [24] reported on an increased EMG signal during voiding in more than 50% of 321 female patients with predominant stress urinary incontinence and no signs for a neurological disorder. In addition, we previously found an elevated EMG signal during detrusor contraction in 71% (30/42) of healthy volunteers [5]. Considering these results, it should be taken into account that a VUDI is not a physiological examination. Indeed, the examination itself can lead to a reflex contraction through irritations by the transurethral catheter. The placement of intramural sphincter needle electrodes as described by Blaivas et al. [8] might have given more insights ruling out non urethral and/or periurethral striated pelvic floor muscle activities potentially allowing more distinguished analyses, particularly in combination with neurophysiological recordings. However, the placement of needle electrodes can be difficult and painful, especially in persons with intact sacral sensation.

We focused in the present study on EMG-based DSD classifications but only included individuals with both an EMG-based and fluoroscopically confirmed DSD (see EMG and fluoroscopic findings in Fig. 2a, b). Combined pelvic floor EMG and fluoroscopy during VUDI are the most accepted and widely agreed methods for diagnosing DSD. Concordance of DSD between EMG and VCUG is reported to be 60% [25].

### Implications for practice

The diagnosis of DSD is made during (V)UDI by EMG, fluoroscopy and pressure-flow study. Combined diagnostic modalities can improve detection of DSD [26]. Nevertheless, the interpretation of a VUDI should in general be made in considerations of the patient’s symptoms [18, 19]. This can be particularly challenging in individuals with an impaired awareness of the LUT after SCI or due to spina bifida. Due to the lack of a DSD-based risk stratification for potential upper urinary tract deterioration (i.e., maximum storage detrusor pressure >40cmH2O) an aggressive therapeutic pathway and a close urodynamic follow-up seems mandatory in these patients [27]. In individuals without diagnosed neurological disorder, the observation of a DSD, especially in concurrence with detrusor overactivity, should be evaluated carefully. In case of any doubt, these individuals should be referred to a neurologist for further evaluation of a potential underlying neurological condition.

The management of DSD remains a challenge since no causal but only symptomatic therapies exist. Some patients can be managed by surveillance. However, in case of LUT symptoms impairing patient’s quality of life, relevant post-void residual, or high intravesical storage pressures, a therapy should always be considered regardless of the etiology. Importantly, even if the risk for lower and upper urinary tract damage, eventually resulting in end-stage renal disease, is highest in individuals with SCI or spina bifida [6], absence of an underlying neurological disease (e.g., for the patient presented in Fig. 2b) does not prevent from potential renal damage as for example in conditions as the Hinman syndrome. These patients should be treated and followed-up as rigorously as individuals with SCI.

### Implications for research

Neurophysiology is considered more sensitive than neuroimaging in evaluation of spinal tract damage as it does not only assess for morphological, but more importantly for functional deterioration [16, 17, 28]. However, normal clinical findings do not exclude underlying pathologies. A long-term evaluation of repeated neuro-urological examinations, VUDI and neurophysiological examinations would be of upmost importance to evaluate if a pathological finding during VUDI could be an early sign of a neurological disease such as multiple sclerosis, spinal canal stenosis, tethered cord syndrome or others. In the future, other neurophysiological examinations particularly designed for the LUT could give further insights regarding underlying pathologies [29, 30].

### Limitations of the study

Although we evaluated a well-defined population with DSD, there are limitations that should be addressed. Our department is part of a highly specialized university SCI center. A negative selection bias, i.e., inclusion of more severe cases, cannot be completely ruled out. Nevertheless, the present study was representative of our daily clinical practice. We did use very strict inclusion criteria based on clinical, neurophysiological and VUDI findings. This led to a rather small cohort. However, the two study groups were well-defined and rigorously examined regarding neurogenic and non-neurogenic LUTD to maximize the chance to detect relevant findings. In addition, a longitudinal follow-up of our study cohort would be highly warranted since it could provide further insights regarding potential risk factors for upper urinary tract damage in the long-term.

## Conclusions

None of the investigated DSD definitions can distinguish between patients with SCI and with NO ND. The more complex DSD classifications by Yalla, Blaivas or Weld cannot compete with the ICS binary yes-no definition which is pragmatic and straightforward. Nevertheless, to allow an appropriate and patient-centered management of individuals with LUTD urodynamic observations have to be evaluated in the context of symptoms and clinical findings.

## Data Availability

For researchers who provide a methodologically sound proposal individual participant data that underlies the results reported in this article, will be available from the corresponding author [TMK] on request, after de-identification. Data will only be provided to achieve the aims in the approved proposal.
